# A Modified Chronic Infection Model for Testing Treatment of *Staphylococcus aureus* Biofilms on Implants

**DOI:** 10.1371/journal.pone.0103688

**Published:** 2014-10-03

**Authors:** Nis Pedersen Jørgensen, Rikke Meyer, Frederik Dagnæs-Hansen, Kurt Fuursted, Eskild Petersen

**Affiliations:** 1 Department of Infectious Diseases, Institute of Clinical Medicine, Aarhus University Hospital, Aarhus, Denmark; 2 Department of Clinical Microbiology, Aarhus University Hospital, Aarhus, Denmark; 3 Interdisciplinary Nanoscience Center (iNANO), Aarhus University, Aarhus, Denmark; 4 Department of Biomedicine, Faculty of Health Sciences Aarhus University, Aarhus, Denmark; 5 Microbiology and Infection Control, Statens Serum Institut, Copenhagen, Denmark; Rockefeller University, United States of America

## Abstract

Bacterial biofilms causing implant-associated osteomyelitis is a severe complication with limited antimicrobial therapy options. We designed an animal model, in which implant associated osteomyelitis arise from a *Staphylococcus aureus* biofilm on a tibia implant. Two bioluminescently engineered (*luxA-E* transformed), strains of *S. aureus* were utilized, Xen29 and Xen31. Biofilm formation was assessed with epifluorescence microscopy. Quantitative measurements were performed day 4, 6, 8, 11 and 15 post-surgery. Bacteria were extracted from the biofilm by sonication and the bacterial load quantified by culturing. Biofilm formation was evident from day 6 post-implantation. Mean bacterial load from implants was ∼1×10^4 ^CFU/implant, while mean bacterial load from infected tibias were 1×10^6 ^CFU/bone. Bioluminesence imaging revealed decreasing activity throughout the 15-day observation period, with signal intensity for both strains reaching that of the negative control by day 15 while there was no significant reduction in bacterial load. The model is suitable for testing antimicrobial treatment options for implant associated OM, as treatment efficacy on both biofilm and viable counts can be assessed.

## Introduction

The incidence of implant-associated osteomyelitis (OM) is increasing, with approximately 100,000 cases of OM each year in the United States of America, and an infection rate of 5–15% in fracture-fixation devices and 0.3–1% in joint-prosthesis [1]–[3]. Staphylococci are the most common bacteria isolated in implant-associated OM, with *Staphylococcus aureus* accounting for 35% and coagulase-negative Staphylococci for 40% [1]. Infected implants colonized by biofilm-forming bacteria are very difficult to diagnose and treat effectively [4].

Biofilms are communities of sessile bacteria enclosed in an extracellular matrix. Compared to the planktonic phenotype, bacteria of the biofilm phenotype are resistant to the host immune response and to antimicrobial treatment [2]–[3]. The increased resistance is partly caused by a lower metabolic rate in biofilm bacteria, and the embedding of bacteria in an extracellular polymer matrix [4]–[7]. The matrix of staphylococcal biofilms consists of a variety of different molecules, such as exopolysaccharides, proteins and extracellular DNA [8], [9]. Besides increasing resistance against antibiotics, the matrix confers mechanic stability and ensures the close proximity between bacteria, allowing cell-to-cell communication and exchange of genetic material, both of which is vital to the biofilm [10], [11].

While the majority of our current knowledge on biofilms stem from *in vitro* studies, animal models are in high demand to investigate the clinical aspects of biofilm infections. A murine model has been proposed using bioluminescence imaging (BLI) and quantitative PCR as quantitative endpoints for the bacterial load [12]. This model employs a PCR based detection of bacterial load from infected bone, but does not address the vital issue of biofilm formation on the implant. In addition, the bacterial load from the implant itself is not addressed and bacterial viability remains unknown. By utilizing a similar surgical procedure as described by Li [12], we have established a novel model in which the presence of an implant-associated biofilm is confirmed and evaluated by combining *in vivo* monitoring and end-point quantification of viable cells in the biofilm. We monitor bacterial metabolic activity *in vivo* by bioluminescent imaging (BLI), and quantify bacterial load at specific time points by culturing from both the implant and infected bone. Finally, the biofilm was visualized on the implant by fluorescence microscopy. Biofilm formation on the implant was induced by preconditioning the implant in murine serum and subsequently incubated it in a culture of *S. aureus* for 24 h, which is sufficient to allow biofilm formation.

## Materials and Methods

### Generation of pathogenic challenge

Stainless steel insect pins with a diameter of 0.25 mm (Benfidan, Nykøbing Mors, Denmark) were used as tibia implants. The pins were autoclaved and stored in 70% ethanol. The pins were placed in 50 ml phosphate buffered saline (PBS) containing 25% murine serum at 37°C for 24 hours to create a conditioning film of matrix molecules. Pins were then transferred to 50 ml Müeller-Hinton broth (SSI Diagnostic, Hillerød, Denmark) with 3.5% NaCl and approximately 1×10^6^ Colony Forming Units (CFU)/ml *S. aureus*, incubated for 24 hours at 37°C, and then stored at −80°C until further use. Two strains of bioluminescent *S. aureus* containing the *luxABCDE* gene as a chromosomal insert were used: The methicillin-susceptible Xen29 strain, and the methicillin-resistant Xen 31 (Caliper, Alameda, CA). The initial bacterial load was measured immediately after incubation prior to storage at −80°C prior to inserting the pin into the animals was 3.2±2.1×10^4 ^CFU/pin for Xen31 and 1.7±5.1×10^4 ^CFU/pin for Xen29.

### Murine model and animal surgery

All animal studies were performed under protocols approved by the Animal Research Inspectorate under the Danish Ministry of Justice (Permission number 2005/561-1071 and 2012-15-2934-00716). The surgical procedure in the model developed here was based upon a previously described procedure in an animal model of chronic OM^12^, in which a steel pin colonized with *S. aureus* induced OM in the tibia of a C57Bl/6 mouse.

Female C57Bl/6 mice, 6–8 weeks old were anaesthetized with isoflurane (2-chloro-2-(difluoromethoxy)-1,1,1-trifluoro-ethane) inhalation. The skin above the tibia was disinfected using 70% ethanol. The metaphysis of the tibia was approximated by palpation. The pin was then implanted transcortically through the metaphysis. Afterwards, the pin was bent on both ends and cut adjacent to skin, to secure it in the bone. The mice received a subcutaneous injection of 0.15 mg/kg Buprenorphine (Temgesic, Schering-Plough, Brussels, Belgium) before they were returned to their standard isolator cages.

The study included 65 animals divided in two groups of 30, which were infected with either Xen29 or Xen31 and 5 animals receiving sterile pins as negative controls. Euthanization of five animals in each group was scheduled to day 4, 6, 8, 11 and 15 after surgery. Five animals from each group were kept in separate cages and were utilized for bioluminescence imaging (BLI). The animals were anaesthetized with isoflourane, intracardial aspiration yielded blood for culture and the mice were then euthanized by cervical dislocation. The tibias were removed postmortem, and were immediately frozen at −80°C. Blood was drawn antemortem by intracardial aspiration, with a total volume of 0.3 ml drawn from each animal. Blood cultures were done on 5% blood agar plates (SSI Diagnostic, Hillerød, Denmark) and incubated for 24 hours in 37°C. Three mice, with sterile pins implanted, served as negative controls and were euthanized on day 15 by cervical dislocation, upon which the tibias and pins from control animals were frozen at −80°C for subsequent analyses.

### Bacterial counts - tibia samples

Tibia samples had the pins carefully extracted from the infected tibia after which the shaft of the bone was removed using a scalpel. The feasibility of bone homogenization and extraction of viable cells from small animals have previously been demonstrated [13]. The remaining tibia segment was cut into smaller sections, and the fragments from each bone where collected individually in Precellys MK28 tubes (Bertin Technologies, Saint Quentin, France) containing steel beads. To ensure standardization of the tibia fragments, the fragments were weighed after the shaft was removed (mean weight 0.08 g±0.01 g). Tubes with tibia fragments were stored in −20°C for 1 hour before addition of 0.5 ml cold (5°C) PBS and immediate transfer to a bead beater (Precellys 24 tissue homogenizer, Bertin Technologies). Homogenization of the tibia was achieved by running a 2×20 seconds 5000 rpm sequence (mean sample temperature 28.3°C, range 27.7–29.1°C) followed by 10 min storage at −20°C and an additional 2×20 second 5000 rpm sequence mean (sample temperature 28.9°C, range 28.5–29.2°C). This yielded a nearly fully homogenized liquid sample. Serial 10-fold dilution in isotonic saline was performed on each sample and 10 µl of each dilution was seeded on 5% blood agar plates and incubated for 24 h at 37°C before counting CFU.

To investigate the effect of both homogenization and freezing on the viability of *Staphylococcus aureus* 9 sterile bones were cut into small fragments, then homogenized and spiked with bacteria. Serial dilutions and culturing was performed. Samples were then frozen at −80°C for 60 m and thawed. Immediately afterwards, serial dilutions and culturing was performed. Samples were then homogenized again followed by serial dilutions and culturing. N = 3 for each initial bacterial inoculum.

### Bacterial counts - implant samples

Ultrasonication was utilized to release bacteria in biofilms on the surface of the pin [14]. The pins were placed in Eppendorff tubes, containing 0.5 ml PBS were then individually vortexed using a Vortex Genie for 30 seconds. The tubes were then placed in an ultrasonic bath (USCS1700 T, VWR, Westchester, PA, USA) and sonicated at 45 kHz and 110 W for 5 minutes. Ultrasonication at this frequency and duration has previously been demonstrated not to affect the viability of *Staphylococcus aureus* [15]. Each tube was then vortexed for 30 seconds and serial dilution was performed and 10 µl of each dilution was seeded on 5% blood agar plates and incubated for 120 h at 37°C before counting CFU.

### BLI, epifluorescence microscopy and confocal laser scanning microscopy

Bioluminescence from the infected tibia was measured on day 4, 6, 8, 11 and 15 with an IVIS Lumina II camera system (Caliper Life Sciences, Massachusetts, USA), using 2 minutes high sensitivity imaging on a defined region of interest with a diameter of 1.5 cm as previously described [16]. Bioluminescence was measured as total flux in P/sec. Animals were euthanized as described above after measuring the bioluminescence on day 15, and the pins were extracted from the tibia. Pins were washed in 50 uL saline to remove lysed leukocytes and other components of the immune response before visualizing *S. aureus* biofilms on the surface of the extracted pins, which were stained with 2 µl 1‰DAPI (4′,6-diamidino-2-phenylindole) and imaged using a Zeiss Axiovert 200 M epifluorescence microscope (Carl Zeiss AG, Oberkochen, Germany) Live/dead stain was made by mixing component A and component B in a 1∶1 ratio (Component A; SYTO 9 dye, 1.67 mM/Propidium iodide, 1.67 mM, Component B; 300 µL solution in DMSO and SYTO 9 dye, 1.67 mM/Propidium iodide, 18.3 mM, 300 µL solution in DMSO).

### Treatment study

A separate treatment study was performed; 10 animals were observed for 11 days after implantation of Xen 29 colonized pins. Antibiotic treatment was initiated on day 12, with 5 animals receiving vancomycin 180 mg/kg s.c q12 and 5 animals receiving saline. Vancomycin is usually administered as 110–180 mg/kg twice daily in murine infection models [17], [18]. We chose 180 mg/kg since Xen 29 vancomycin minimal inhibitory concentration is 2 µg/ml (Etest) and serum monitoring of vancomycin concentration levels are unfeasible in murine models.

Total treatment duration was 14 days, after which animals were euthanized, and samples collected and processed as described under bacterial counts - implant samples and bacterial counts - tibia samples.

*Statistical analysis:* All statistical calculations were performed with STATA 12.1 (STATACORP, Texas, USA) and figures were made with GraphPad Prism 5.04 (Graphpad Software, California, USA).

## Results

### Animal welfare

Throughout study 1 and study 2 both weight and activity levels were monitored to ensure that the ongoing OM did not have any adverse effects on the overall health of the test animals. We found no significant reduction in animal weight (data not shown) and activity levels did not decrease (data not shown). All blood cultures were negative (data not shown).

### Bacterial recovery unaffected by homogenization

Sterile bones spiked with bacteria demonstrated a reduction in 0.4–0.9 log from being frozen at −80°C for 60 minutes, 5.4 log CFU/ml (range: 5.3–5.4 log CFU/ml) to 4.5 log CFU/ml (range: 4.2–4.6 log CFU/ml) (P = 0.05, Mann-Whitney test) and 3.7 log CFU/ml (range 3.7–4.0 log CFU/ml) to 3.3 log CFU/ml (range: 3.2–3.3 log CFU/ml) (P = 0.0388, Mann-Whitney test. The homogenization did not affect bacterial viability, as median log CFU/ml did not differ between freezing compared to freezing + homogenization; 4.5 log CFU/ml, range 4.2–4.6 log CFU/ml vs 4.5 log CFU/ml, range 4.4–4.6 log CFU/ml and 3.3 log CFU/ml, range 3.2–3.3 log CFU/ml vs 3.2 log CFU/ml, range 3.2–3.3 log CFU/ml.

### Biofilm formation by day 6 post surgery with continued viability throughout observation period

**Figure 1:**
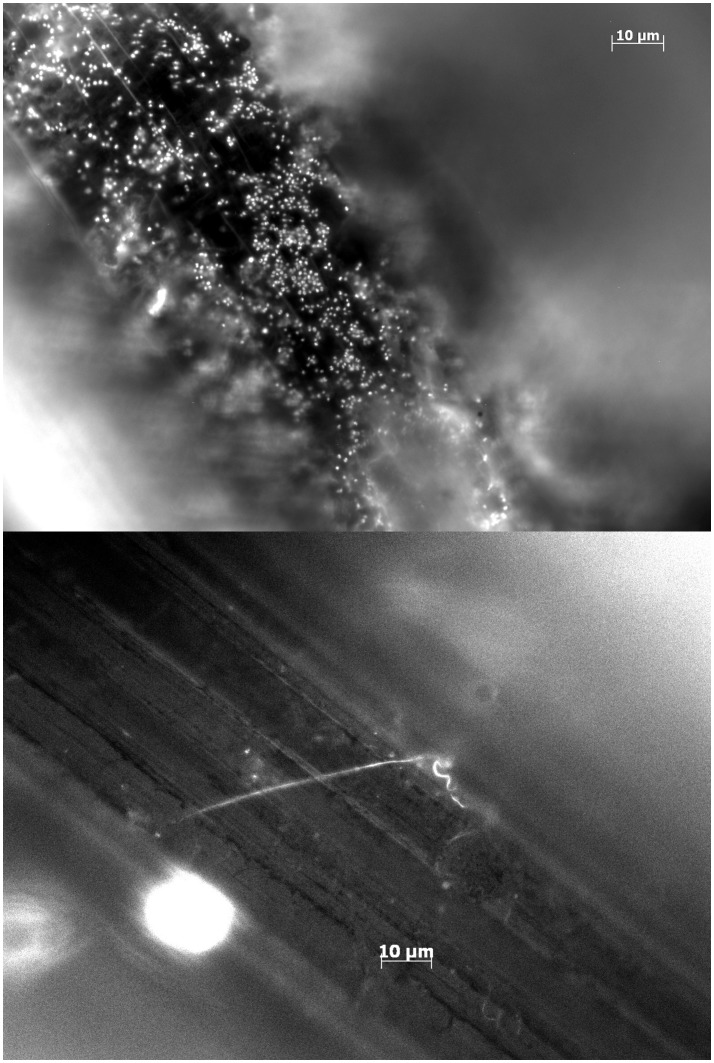
Biofilm assesment with epifluorescence microscopy. Figure 1A shows Xen29 biofilm formation by day 6 post implantation. Implants are washed in saline and stained with DAPI. Along the surface of the implant, clusters of coccoid cells with a diameter of 1 µm are found. The morphology of these cells are consistant with *Staphylococcus aureus.* Figure 1B shows a sterile implant 14 days post implantation.

**Figure 2:**
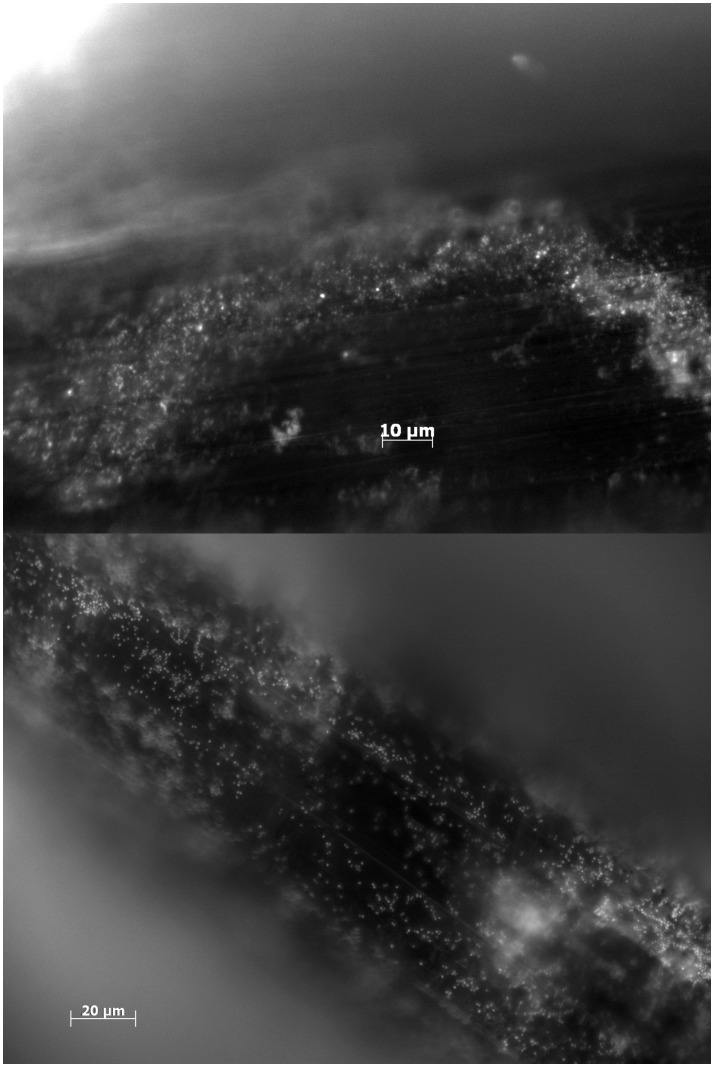
Biofilm assessment with epifluorescence microscopy. Figure 2A and 2B show biofilm formation by day 8 from two different samples. The biofilm formation is heterogenous from implant to implant. Implants are washed in saline and stained with DAPI. Biofilm formation by *S. aureus* exhibits intersample variation in both distribution and thickness, but was consistently found.

**Figure 3:**
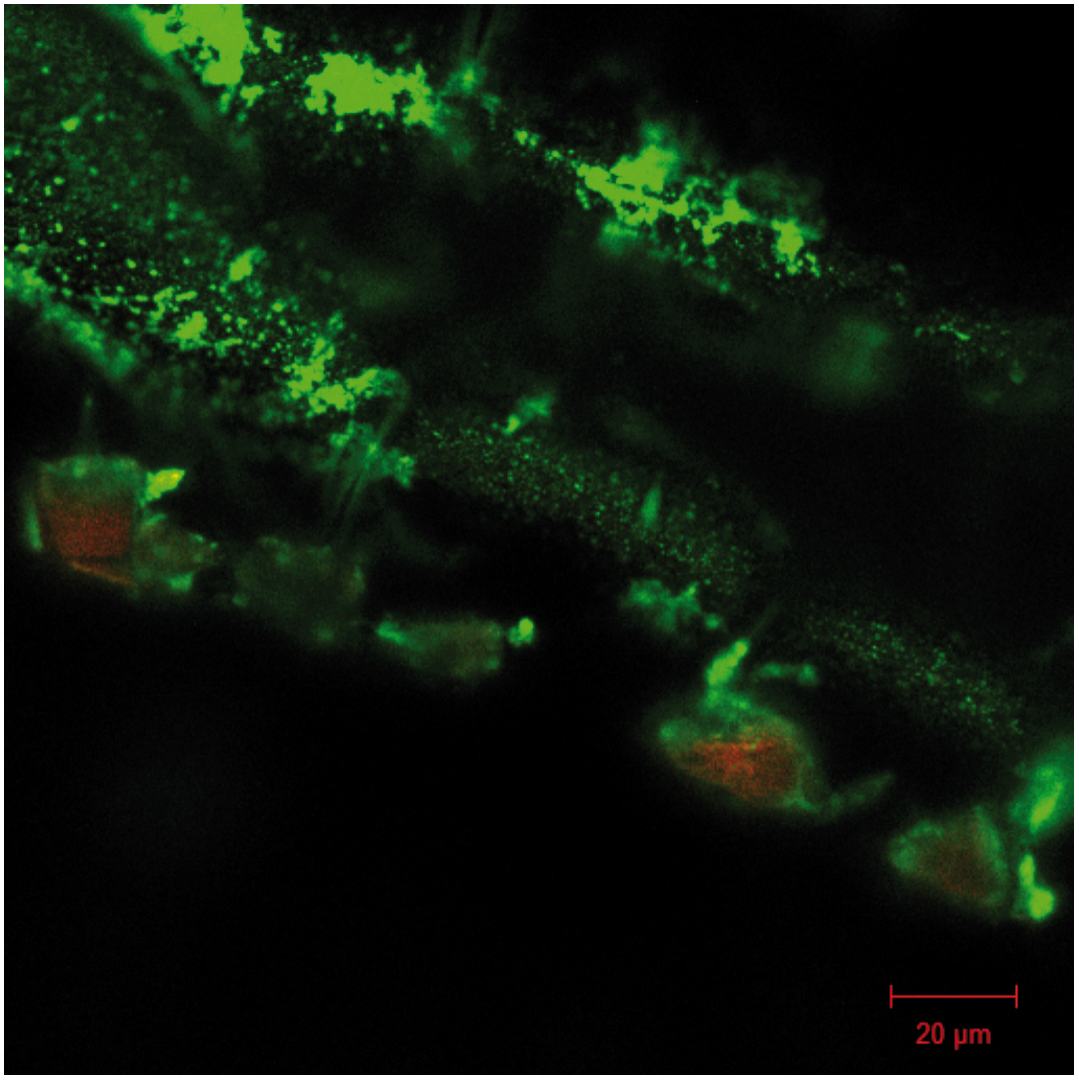
Biofilm assessment with confocal laser scanning microscopy. LIVE/DEAD stain of biofilm formation by day 15 post surgery on tibia implant. Due to the distinct shape of the implant, z-stack construction was not viable, but the confocal image demonstrate multiple areas of multicellular clusters of coccoid cells, that are viable.

Pins from initial pilot studies (to asses the feasibility of our design) were investigated with epifluorescence microscopy to confirm biofilm formation (Figure 1). Since the animals were observed for a minimum of 4 days before being euthanized, biofilm formation from day 1–3 could not be investigated. While structures that bore resemblance to biofilm clusters were observed by day 4, these inconclusive (data not shown). From day 6 and onwards, biofilms were consistently found on pins colonized with either strain. Multilayered clusters of coccoid cells with an approximate diameter of 1 µm were interpreted as being *S. aureus* biofilms (Figure 1a), while sterile pins were devoid of similar cells (Figure 1b) and indeed, *S. aureus* could be cultured after the pins were sonicated. The distribution of the biofilm along the pin was very heterogeneous (Figure 2A and 2B), making quantification by microscopy unfeasible. Confocal laser scanning microscopy was used to confirm the presence of a viable biofilm (Figure 3). Microscopy was instead used to confirm the presence of biofilms, while quantification of cells in the biofilm was done by CFU counts.

### Stabile bacterial load throughout the observation period

**Figure 4:**
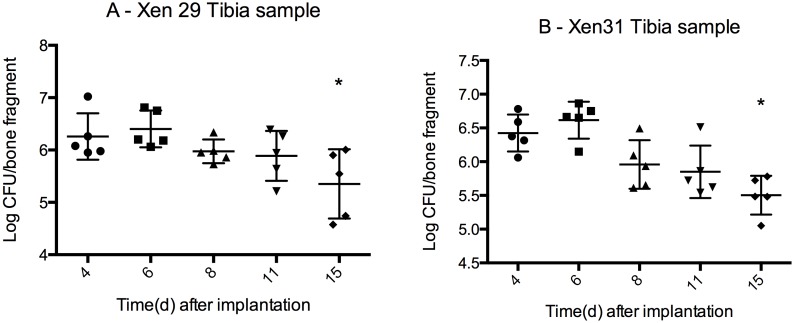
Bacterial load in infected tibia mean CFU±SD (logarithmic scale). Bacterial load from two different *S. aureus* strains, Xen 29(MSSA) and Xen 31(MRSA). The infection is a localized OM adjacent to the stainless steel pin implanted through the metaphysis of the bone. Bone fragment weight 0.08 g±0.01 g. Bacterial load by day 15 are significantly lower (marked with*) than initial load by day 4 for both X29 (P = 0.04) and X31 (P = 0.001). From day 8–15, the infection remains stable (no difference in mean) for both strains (one way ANOVA, P = 0.14 for X29 and P = 0.06 for X31). n = 5 for each group.

Culture was used to quantify the amount of viable bacteria. To extract bacteria from the biofilm and the surrounding infected area, the tibias were homogenized in a bead beater, and bacterial load was measured as CFU/tibia. By day 4 post implantation, OM arose from the colonized pins inserted through the metaphysis with an initial load of 6.1±0.2 log CFU (mean±SD) for X29 and 6.4±0.1 log CFU (mean±SD) for X31 (Figure 4A and 4B.). The initial load was significantly higher than the final load for both strains (X29; 5.4±0.4 log CFU, P = 0.04, t-test and X31; 5.5±0.1 log CFU, P = 0.001, t-test). By day 8, the infection was well established by both X29 and X31, with no significant reduction in CFU from day 8 to day 15 (one way ANOVA, P = 0.14 for X29 and P = 0.06 for X31).

**Figure 5:**
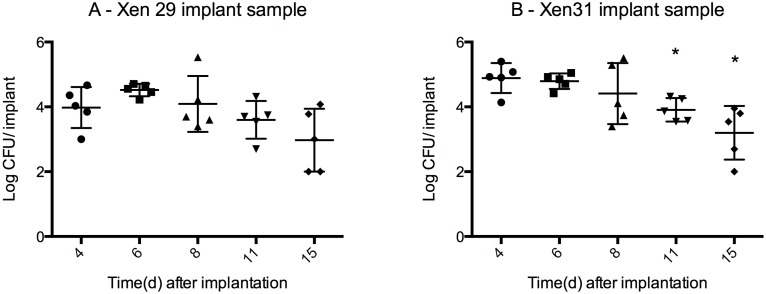
Bacterial load from implants mean CFU±SD (logarithmic scale). The bacteria found in biofilms along the surface of the pin was released with ultrasonication and grown for 24 h at 35C. The pins were washed in saline prior to sonication thus the bacterial load solely represents bacteria from biofilms formed on the surface of the pin. Mean pin weight 0.002±0.0001 g. Approximate bacterial load for all samples is 4 log CFU/implant from day 4–11 for both X29 and X31. The bacterial load is significantly lower for X31 for day 11 (P = 0.006) and 15 (P = 0.004) when compared to day 4, but it remains stable from day 8–15 (no significant change in mean, P = 0.07 one way ANOVA). Xen29 exhibits a similar stabile bacterial load from day 8–15 (no significant difference in means, P = 0.14 one way ANOVA) n = 5 for each group.

Sonication released bacteria from the biofilms formed on the surface of the pins. The bacterial load of both strains on the pins (Figure 5A and 5B) was lower than the load in the bone fragments, with an ∼mean of 4 log CFU/implant from day 4–11 for both X29 and X31. The bacterial load is significantly lower for X31 for day 11 (3.9 log CFU±0.1, mean±SD, P = 0.006, t-test) and 15 (3.2 log CFU±0.7, P = 0.004) when compared to day 4 (4.9 log CFU±0.2), yet it remains stable from day 8–15 (no significant change in mean, P = 0.07 one way ANOVA). Xen29 exhibits a similar stabile bacterial load from day 8–15 (no significant difference in means, P = 0.14 one way ANOVA), yet the initial bacterial load is not significantly higher than the bacterial load by day 11 (P = 0.35, t-test) or day 15 (P = 0.09).

### Decreasing bioluminescent activity over time, while bacterial load remain stabile

**Figure 6:**
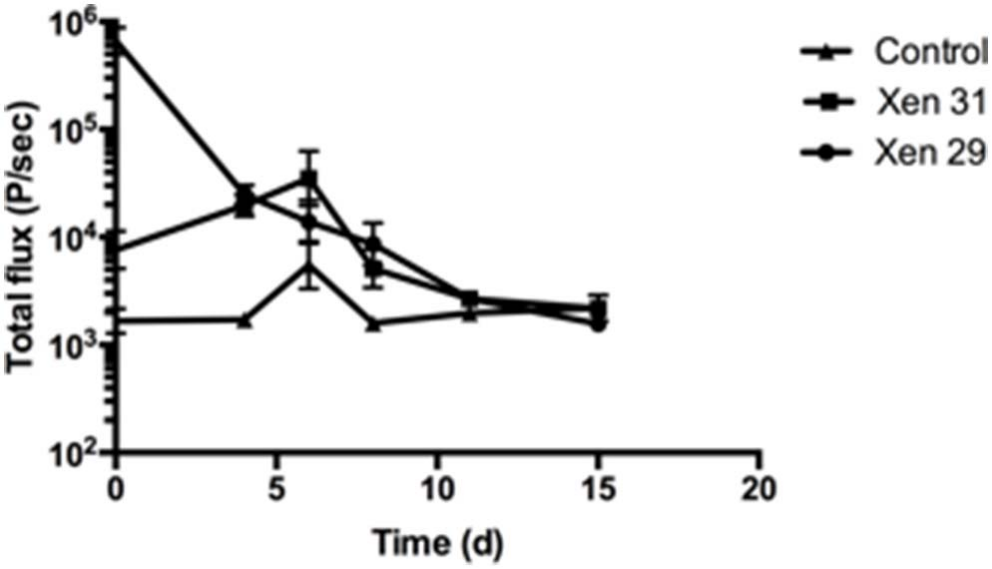
Bioluminescent imaging: mean Total flux (P/sec)± SD for X29, X31 and sterile implant. 2 minutes high sensitivity measurement of 1,5 cm^2^ ROI. Xen 29 demonstrates a steady decrease in total photon flux, from 6.6×10^6^ P/sec to 1.5×10^4^ P/sec by day 15, which correspond to the levels seen in negative controls. Xen30 demonstrated an initial increase in bioluminescence from 7.5×10^4^ to 3.5×10^5^ P/sec from day 0 to 6, followed by a decrease to 5.1×10^4^ P/sec on day 8 and by day 11 it’s signal was comparable to the negative control. While the BLI intensity demonstrated great change over time, bacterial load in the infected tibias (2 logs higher than the bacterial load from the implants and is thus the principal contributor to the BLI signal intensity) remains stabile during day 8–15, in which the BLI intensity from both strains reaches the same intensity as the negative controls. n = 5.

Bioluminescent imaging (BLI) was used to asses total bacterial load in vivo by utilizing strains of genetically altered staphylococci that emit photons when they are metabolically active (Figure 6). A steady decrease in total photon flux was observed for Xen29, from 6.6×10^6^ P/sec to 1.5×10^4^ P/sec by day 15, which correspond to the levels seen in negative controls (sterile implant and uninfected bone). Infection with the Xen30 strain was characterized by an initial increase in bioluminescence from 7.5×10^4^ to 3.5×10^5^ P/sec from day 0 to 6, followed by a decrease to 5.1×10^4^ P/sec on day 8.

### Vancomycin treatment was unable to eradicate biofilm formation

**Figure 7:**
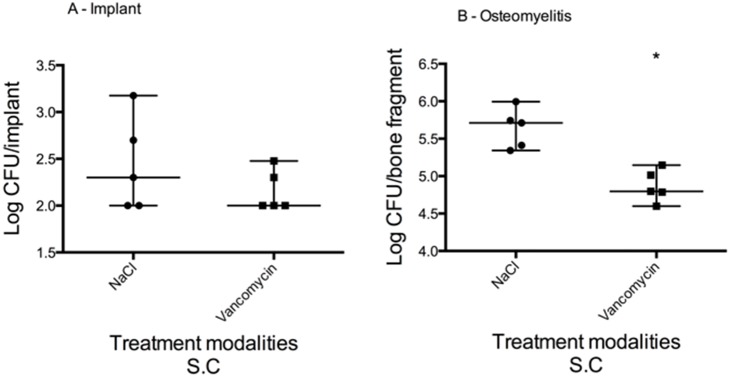
Vancomycin treatment effect on biofilm on implant and osteomyelitis, median and range (logarithmic scale). On day 12 post surgery, vancomycin treatment (180 mg/kg S.C q12) was started. Treatment duration was 14 days. Treatment outcome was evaluated on both implant samples (a) and tibia samples (b). The vancomycin was unable to eradicate the biofilm formed on the surface of the implant, yet it did have an effect on the osteomyelitis. Bacterial load was reduced from 5.7 (range: 5.4–5.9) log CFU/bone fragment to 4.8 (range: 4.6–5.1) log CFU/bone fragment (Mann-Whitney, P = 0.0079).

Despite a 14-day treatment of vancomycin, the biofilm on the surface of the implant persisted (Figure 7a). The bacterial load on the implants from animals treated with vancomycin was not statistically different to the bacterial load on the implants from animals treated with NaCl. In comparison, the vancomycin had an effect on the osteomyelitis, reducing the bacterial load from 5.7 (range: 5.4–5.9) log CFU/bone fragment to 4.8 (range: 4.6–5.1) log CFU/bone fragment (Mann-Whitney, P = 0.0079) (Figure 7b).

## Discussion

Foreign body related biofilm infections represent a disastrous complication for patients. Treatment options are currently limited, in part due to the lack of reliable animal models to further investigate antimicrobial treatment options from *in vitro* studies. Animal models are important when investigating implant associated OM, because they allow for control of key parameters to the overall treatment outcome: host immune system, biofilm colonization of implants and infection in the adjacent bone, altered diffusion of antimicrobials into infected tissue etc. Such parameters must be taken into account when assessing treatment efficacy. A final issue is the lack of understanding between infection duration and biofilm maturity. Clinical experience with biofilm associated foreign body infections points towards an inverse correlation between duration of infection and treatment outcome, and the “mature” biofilm infections are virtually untreatable with conventional antimicrobial therapies [19].

We designed a model with biofilm formation on the implant surface and an infection in the adjacent bone, with a long observational period and no overall decline in animal welfare. Our model was developed from a previous model, in which the pathological hallmarks of chronic OM had been established. An important aim of this study was to verify when a biofilm was established on the implant, as studies of biofilm treatment strategies should not commence before this point. Also the biofilm and OM should not be cleared by the murine immune system, as that would compromise the possibility of assessing treatment efficacy. Many models of implant associated infections caused by biofilm formation [20], [21] start treatment 3–72 hours after initiating infection without a prior examination of actual biofilm formation. Our treatment study demonstrated that vancomycin treatment did reduce the bacterial load in the infected tibia, there was no effect of the treatment on the *S. aureus* biofilm on the surface of the implant. It is also interesting to note that the vancomycin treatment did significantly reduce the bacterial load in the infected bone, this reduction was only approximately 1 log CFU. This could be due to insufficient treatment duration or poor vancomycin penetration into infected bone.

The location and volume of biofilm differed from pin to pin, which is most likely due to biological variation, but consistent biofilm formation was seen by day 6 post implantation. The actual timepoint, at which the biofilm is formed, and the different stages of maturation were not ascertainable with our setup. By day 6 biofilms were consistently found, yet that does not exclude the possibility, that these are formed much sooner.

DNA based techniques for bacterial identification is rapidly becoming a routine investigation in clinical microbiology departments, yet viability of bacteria by culturing remains a desirable method in animal models designed for antimicrobial efficacy testing, as this is better suited for assessing treatment efficacy and potential changes in antibiotic susceptibility following treatment. With tissue homogenization by a bead beating, we were able to extract viable bacteria from the infected tibia. While some viability was lost due to freezing, the homogenization itself did not affect bacterial viability. The small standard deviation on the results implies high reproducibility of the method. The major obstacle with tissue homogenization is the amount of energy required to liquefy solid tissue, which potentially increases temperature within the samples to levels. To alleviate this, we chose young animals, aged 6–8 weeks, with cartilaginous bones, and as the OM was localized in the trabecular metaphysis, we removed the shaft of the tibia, which consists primarily of compact osseous tissue, and thereby reduced bead beating time and intensity.

The bacterial load from both bone and implant were comparable to findings from previous studies using a model of post arthroplasty infection. By injecting 5×10^4 ^CFU, Bernthal et al [22] were able to induce a joint infection in relation to a foreign body. The bacterial load from implant, joint fluid and infected soft tissue was approximately 6 log CFU 5 days after inoculation, which is comparable to the bacterial load found in our model.

The BLI measurements demonstrated that the bioluminescent activity of both strains decreased during the study, despite the stable bacterial load. This observation was surprising, as BLI measurements are frequently used in animal models of implant associated infections, and the bacterial load in our study is of such a magnitude that a BLI signal should be measureable [16], [22], [23]. A repeat study with new animals confirmed the results. By comparing our study design with other studies^12,16^, the most likely explanation is that we used an increased incubation period of the pins prior to implantation. In most studies with BLI measurements [12], [16], [18], the bacteria were brought into log phase prior to implantation or injected as planktonic bacteria in relation to a foreign body. In our model, we used a 24 hour incubation period in broth preceded by a preconditioning phase in 25% murine serum which could be an explanation for the decrease in bioluminescent intensity (Kevin Francis, personal correspondence). A model of chronic, medullar OM [24] demonstrated a similar decrease in BLI intensity over time, yet at different time points, which is most likely due to variation in model design. BLI measurements are a useful tool when investigating in vivo infections, but must always be accompanied by other quantitative measurements and it must be taken into account, that for Xen 29 and Xen 31 the exact promotors for the *luxABCDE* gene are unknown and the bioluminescent signal is oxygen dependent, which decreases it’s applications in anoxic conditions.

In conclusion, we have modfied a murine model to further its usage in investigating implant-associated osteomyelitis. This model has a high infection rate (>95%), the infection is is not cleared by the murine immune response and the infection is not systemically disseminated. Vancomycin was unable to eradicate the biofilm infection, despite 14 days treatment. This further validates that this model accurately mimics a chronic, implant-associated osteomylitis.
