# Correction: A Modified Chronic Infection Model for Testing Treatment of *Staphylococcus aureus* Biofilms on Implants

**DOI:** 10.1371/journal.pone.0118124

**Published:** 2015-03-06

**Authors:** The PLOS ONE Staff

The following information is missing from the Funding section: “Additional funding from Pfizer Investigator-initiated research (IIR) Grant, 2009 & Pfizer Antibacterial EUROPE ASPIRE 2010.”

The complete, correct Funding section is: “Nis Jørgensen is supported by a PhD grant from the Faculty of Health Science, University of Aarhus, Denmark. Additional funding from Pfizer Investigator-initiated research (IIR) Grant, 2009 & Pfizer Antibacterial EUROPE ASPIRE 2010. The funders had no role in study design, data collection and analysis, decision to publish, or preparation of the manuscript.”

Additionally, the second author’s middle initial is missing. The full name for this author is: Rikke L. Meyer. The correct citation is: Jørgensen NP, Meyer RL, Dagnæs-Hansen F, Fuursted K, Petersen E (2014) A Modified Chronic Infection Model for Testing Treatment of Staphylococcus aureus Biofilms on Implants. PLoS ONE 9(10): e103688. doi:10.1371/journal.pone.0103688
